# The Mechanical Effect of the Periodontal Ligament on Bone Strain Regimes in a Validated Finite Element Model of a Macaque Mandible

**DOI:** 10.3389/fbioe.2019.00269

**Published:** 2019-10-30

**Authors:** Hyab Mehari Abraha, Jose Iriarte-Diaz, Callum F. Ross, Andrea B. Taylor, Olga Panagiotopoulou

**Affiliations:** ^1^Moving Morphology and Functional Mechanics Laboratory, Department of Anatomy and Developmental Biology, Monash Biomedicine Discovery Institute, Monash University, Clayton, VIC, Australia; ^2^Department of Biology, The University of the South, Sewanee, TN, United States; ^3^Department of Organismal Biology and Anatomy, University of Chicago, Chicago, IL, United States; ^4^Department of Basic Science, Touro University, Vallejo, CA, United States

**Keywords:** finite element analysis, *in vivo* validation, biomechanics, mastication, rhesus monkey, sensitivity analysis

## Abstract

The primary anatomical function of the periodontal ligament (PDL) is to attach teeth to their sockets. However, theoretical and constitutive mechanical models have proposed that during mastication the PDL redistributes local occlusal loads and reduces the jaw's resistance to torsional deformations. These hypotheses imply that accurately modeling the PDL's material properties and geometry in finite element analysis (FEA) is a prerequisite to obtaining precise strain and deformation data. Yet, many finite element studies of the human and non-human primate masticatory apparatus exclude the PDL or model it with simplicity, in part due to limitations in μCT/CT scan resolution and material property assignment. Previous studies testing the sensitivity of finite element models (FEMs) to the PDL have yielded contradictory results, however a major limitation of these studies is that FEMs were not validated against *in vivo* bone strain data. Hence, this study uses a validated and subject specific FEM to assess the effect of the PDL on strain and deformation regimes in the lower jaw of a rhesus macaque (*Macaca mulatta*) during simulated unilateral post-canine chewing. Our findings demonstrate that the presence of the PDL does influence local and global surface strain magnitudes (principal and shear) in the jaw. However, the PDL's effect is limited (diff. ~200–300 με) in areas away from the alveoli. Our results also show that varying the PDL's Young's Modulus within the range of published values (0.07–1750 MPa) has very little effect on global surface strains. These findings suggest that the mechanical importance of the PDL in FEMs of the mandible during chewing is dependent on the scope of the hypotheses being tested. If researchers are comparing strain gradients across species/taxa, the PDL may be excluded with minimal effect on results, but, if researchers are concerned with absolute strain values, sensitivity analysis is required.

## Introduction

The periodontal ligament (PDL) is a fibrous tissue of varying thickness (0.15–0.38 mm) that attaches the root cementum of each tooth to its surrounding alveolar bone (Berkovitz, 1990; Nanci and Bosshardt, 2006). Previous studies have linked the PDL's complex geometry (Berkovitz, 1990; Nanci and Bosshardt, 2006; Ho et al., 2013) and material properties (heterogeneity, anisotropy, and viscoelasticity) (Andersen et al., 1991a; Van Driel et al., 2000; Dorow et al., 2003, 2014; Fill et al., 2011, 2012; Minch, 2013; Keilig et al., 2016) to its mechanical role in facilitating tooth mobility (Bien, 1966; Provatidis, 2000; Pietrzak et al., 2002; Natali A. N. et al., 2004; Qian et al., 2009; McCormack et al., 2014) and absorbing and re-distributing occlusal loads (Reinhardt et al., 1984; Mohl, 1988; Daegling et al., 1992; Jeon et al., 1999; Rees, 2001; Poiate et al., 2009; Ren et al., 2010; Nikolaus et al., 2017). For instance, in a small-scale finite element analysis (FEA) of an isolated tooth-PDL-bone segment from an adult sow, Nikolaus et al. (2017) found that the PDL modeled with non-uniform thickness increased stresses in alveolar bone compared to models with uniform PDL thickness. From their results (Nikolaus et al., 2017) concluded that (a) the PDL protects the tooth roots by redirecting stresses into the alveolar bone and (b) PDL geometry (thickness) determines how these stresses are distributed across alveolar bone. Similarly, McCormack et al. (2017) showed that modeling the fibrous structures of the PDL (compared to modeling it as a solid object) in a section of a human mandibular corpus increased alveolar bone strains around the tooth socket (>100 με).

While these studies have used small-scale FEMs to highlight the importance of accurately modeling the PDL's geometry, capturing the complex fibrous structure of the PDL in large-scale specimens (e.g., whole mandibles or crania) is not possible due to resolution constraints on computed tomography (CT) and μCT scans. Resolution and soft tissue contrast limitations also mean that PDL geometry is typically captured by selecting the space the PDL occupies and not the actual geometry of the tissue (Kupczik et al., 2009; Cox et al., 2011; Panagiotopoulou and Cobb, 2011; Gröning and Fagan, 2012; Panagiotopoulou et al., 2017).

Further problems arise when assigning material properties to the PDL in FEA. Linear elastic models do not capture the true mechanical behavior of the PDL under load, which previous studies have shown to be non-linear (Bien, 1966; Wills et al., 1976; Fill et al., 2012). However, assigning complex (non-linear) material properties to the PDL in FEA is challenging. Viscoelastic models are able to combine time-dependent viscous (i.e., liquid phase) movement with instantaneous elastic, solid-like behavior (Fill et al., 2012) but data on the relationship between viscoelastic response and the structure of the PDL are limited (Natali A. et al., 2004; Qian et al., 2009; Zhang et al., 2014). In addition, time-dependent viscoelastic material properties cannot be assigned to static FEMs (Fill et al., 2012). While hyper-elastic models can be used in static FEMs (Fill et al., 2012), such models do not include the PDL's fluid phase response to tension-compression loading (Fill et al., 2012). Also, hyper-elastic models are described by a stored energy function that is load case and material specific. As such, the material parameters used in hyperelastic models are ideally derived from load case and tissue- specific experimental data. Finally, while multi-phase models allow for the coupling of viscoelastic and hyper-elastic constitutive models to more accurately represent the global behavior of the PDL the lack of sufficient data on the viscoelastic properties of the PDL limits our ability to design these multi-phase models (Fill et al., 2012).

Due to these modeling limitations, in large-scale and static FEMs (e.g., mandible/crania) the PDL is frequently modeled as a linear elastic, homogeneous, isotropic tissue, and assigned a single Young's Modulus (*E*) value (e.g., Slater et al., 2009, 2010; Cox et al., 2011; Panagiotopoulou and Cobb, 2011; Porro et al., 2013; Panagiotopoulou et al., 2017). However, the PDL has both a time- and direction-sensitive response to load (Sakada and Kamio, 1971; Trulsson et al., 1992), and as a result variation in the experimental protocols used to derive its material properties has led to a wide range of published *E-*values (0.07–1,750 MPa) (Andersen et al., 1991b; Dorow et al., 2003, 2014; Genna et al., 2007; Fill et al., 2011). Consequently, an extensive range of *E*-values has been assigned to the PDL in FEA studies (e.g., Kupczik et al., 2009; Panagiotopoulou and Cobb, 2010; Cox et al., 2011; da Silva et al., 2011; Porro et al., 2013), the effects of which on strain results remains unclear.

Other studies exclude the PDL entirely from large-scale FEMs of the cranium or mandible (Dumont et al., 2005, 2009; Strait et al., 2005, 2007, 2009; Wroe et al., 2007; Slater et al., 2010; Bright and Rayfield, 2011; Bright, 2012; Figueirido et al., 2014; Fitton et al., 2015; Smith et al., 2015a,b; Toro-Ibacache and O'Higgins, 2016; Toro-Ibacache et al., 2016). However, sensitivity analysis of the effects of excluding the PDL in whole FEMs has yielded conflicting mechanical results (Gröning et al., 2011, 2012; Wood et al., 2011; Gröning and Fagan, 2012; Grosse et al., 2012). An early study by Daegling et al. (1992) investigating the influence of the PDL on the mechanics of the mandible proposed that inclusion of the PDL in FEMs, even when modeled with simplicity may affect the transmission of torsional shear stresses from the bone-tooth interface into the alveoli. Researchers also proposed that the presence of the PDL would reduce the torsional rigidity of the jaw (defined as a structure's ability to resist deformations brought about by twisting moments; Daegling et al., 1992). As a decrease in torsional rigidity would constitute decreased resistance to both axial and shear strains (Biewener et al., 1992), it follows that including the PDL in large scale FEMs of the crania or mandible would result in an increase in principal and shear strains across the jaw. A later study of the *Macaca fascicularis* mandible found that FEMs that model bone and teeth as continuous structures (i.e., exclude PDL) report strains lower than those found *in vitro*, lending partial support to the hypothesis that the PDL decreases overall rigidity of the jaw (Marinescu et al., 2005). However, their study lacked a FEM that included the PDL for comparison. More recently, Gröning et al. (2011) FEA of a human mandible showed that inclusion of the PDL increased principal strains in the whole mandible, with ≥500 με increase in areas in the mandibular corpus. The authors also recorded an increase in principal strains inferior to the constrained tooth and proposed that inclusion of the PDL in FEA reduces the stiffness of the human mandible (Gröning et al., 2011). In a follow-up sensitivity analysis, Gröning et al. (2012) found that inclusion of the PDL in a different FEM of the human mandible increased strain magnitudes in the mandibular corpus by more than 40%. By contrast, Wood et al. (2011) tested the effect of the PDL on deformations of a *Cebus* (now *Sapajus*) *apella* cranium using FEA and found that when modeled as an isotropic, homogeneous and linear elastic tissue, inclusion of the PDL had a localized effect on von Mises stress in alveolar bone but only subtle effects on von Mises stress elsewhere in the cranium, even after varying the PDL material properties (linear elastic, hyper elastic, and viscoelastic).

The divergent findings of Gröning et al. (2011) and Wood et al. (2011) have been attributed to a variety of factors, including differences in species (brown capuchin monkey vs. modern human), skeletal structure being modeled (cranium vs. mandible), the size of the tooth root relative to the skeletal structure (teeth to maxilla vs. teeth to mandible), and boundary conditions in the FEA (direct constraint of condyles vs. constraining temporomandibular joint modeled as a layer of soft tissue; Gröning and Fagan, 2012; Grosse et al., 2012). Importantly, however, to our knowledge, no study has assessed the sensitivity of various modeling conditions of the PDL using a subject-specific FEM, validated against *in vivo* bone strain data. Considering the assumptions made when modeling the PDL as a geometrically simplified, homogeneous and isotropic tissue in static, large scale FEMs, it is important to test whether such modeling assumptions affect bone strain results from FEA and whether they yield bone strain results close to *in vivo* bone strain data.

To this end, our study investigated the mechanical influence of the PDL on strain regimes in a validated static FEM of a female rhesus monkey (*Macaca mulatta*) during simulated unilateral post-canine chewing. We tested two hypotheses:

*Hypothesis 1:* Inclusion of the PDL (and associated alveolar tissues), creating a more compliant interface between tooth and bone, decreases torsional rigidity of the jaw and increases global surface strain magnitudes (surface strains across the entire jaw). Exclusion of the PDL (and associated alveolar tissues) increases the jaw's torsional rigidity and decreases global surface strains (Daegling et al., 1992; Gröning et al., 2011). Based on this hypothesis we made the following three predictions:

1. When the PDL is included in FEMs of the jaw, progressive increases in its Young's Modulus (i.e., decreases in its compliance) will increase the torsional rigidity of the jaw, resulting in progressively lower global surface principal strain (ε_1_–maximum principal strain and ε_2_–minimum principal strain) magnitudes.
2. The FEM of the jaw that excludes the PDL will experience lower global surface principal strain magnitudes during simulated chewing compared with the FEM that includes the PDL (Gröning et al., 2011).
3. The FEM of the jaw that includes the PDL will experience greater torsion of the balancing- and working-side corpora, and higher associated shear strains, than the FEM that excludes the PDL.

*Hypothesis 2: The PDL acts to redirect masticatory stresses from the tooth roots to the alveolar bone*. We predict that FEMs that exclude the PDL will have higher strains in the tooth roots compared with FEMs that include the PDL (McCormack et al., 2017; Nikolaus et al., 2017).

## Methods

To test our hypotheses we used a previously published FEM of a rhesus macaque mandible (Panagiotopoulou et al., 2017) and validated our FEMs against *in vivo* bone strain data. The details of capturing model geometry, segmentation, and material property assignment for the cortical bone, trabecular tissue, teeth and PDL are described in full elsewhere (Panagiotopoulou et al., 2017).

In brief, the geometry of the macaque mandible was captured using CT scan data and defined in Mimics v17.0 (Materialize, Leuven, Belgium) (Panagiotopoulou et al., 2017). As the PDL itself cannot be visualized in CT, its geometry was demarcated as a continuous, non-uniform space between the tooth root and alveolar bone (spanning 3–6 voxels), and was segmented using a combination of manual and automatic methods (Panagiotopoulou et al., 2017). The FEM was assigned 80 heterogeneous and orthotropic material properties to the cortical bone using data derived from an *ex vivo* experiment using the ultrasound wave technique (Dechow et al., 2017) and a linear theoretical model to relate Young's modulus and Poisson's ratio to the density of the calibrated CT scans (Dechow et al., 2017; Panagiotopoulou et al., 2017). We assigned the data from the theoretical model to our FEMs by using the formula below to define the relationship between grayscale values (GV) of the CT scans, apparent density and parameters of the material properties (*E, v)*:

$$\begin{array}{l} \text{ Poisson's ratio }(v)=\;0.400595880819907+\\ \;-\text{0}.\text{00000913165104192569 }\times \rho \\ \text{Young's Modulus }(E)=-2.7501977952777819\\ \;+0.0082389155811676013\;\times \rho ,\\ \text{ where Density }(\rho )=-0.01979662\;\times \;1.0577433\;\times \text{ GV}. \end{array}$$

Full details of the experimental protocol for material testing and the development of the theoretical model are provided in Dechow et al. (2017) and Panagiotopoulou et al. (2017), respectively. Isotropic and homogeneous elastic material properties were assigned to the bone screws (*E* = 105 000 MPa; *v* = 0.36), teeth (*E* = 24 500 MPa; *v* = 0.49, PDL (*E* = 0.68 MPa; *v* = 0.49), and trabecular tissue (*E* = 10 000 MPa; *v* = 0.3) (Panagiotopoulou et al., 2017). Bone screws were implanted in the mandible to measure three dimensional (3D) rigid body kinematics of the mandible during the post-canine unilateral chewing modeled in this study (Iriarte-Diaz et al., 2011; Ross et al., 2012; Panagiotopoulou et al., 2017).

### PDL Variants

To determine whether the PDL influenced the mechanics of the jaw during post-canine chewing, we compared the original validated FEM (=PDL model) against models that:

- entirely excluded the PDL by assigning it material properties of the surrounding teeth (*E* = 24 500 MPa and *v* = 0.49) (NO PDL Model) (Supplementary Figure S1).
- varied the Young's Moduli between 0.07, 0.18, 13.8, and 1,750 MPa (Models 1–4, respectively) corresponding to the published *E* data experimentally determined from humans and dogs (Thresher and Saito, 1973; Yettram et al., 1977; Takahashi et al., 1980; Andersen et al., 1991a,b; Goel et al., 1992; Cattaneo et al., 2005; Li et al., 2006). The Poisson's ratio of the PDL was kept constant at *v* = 0.49, as previous sensitivity studies showed that varying the Poisson's ratio between 0.45–0.49 had no effect on FEM behavior (Gröning et al., 2011; Wood et al., 2011).

### Loading Models

All FEMs were loaded using subject-specific muscle force vectors that simulated unilateral post-canine chewing. Muscle-force data were derived from an *in vivo* experiment during which the animal was chewing on nuts (almond, cashew, brazil nut, pecan, walnut, seeds). Detailed experimental protocols for the *in vivo* electromyography (EMG) recording and post-processing is similar to the one provided in Panagiotopoulou et al. (2017), when the animal was chewing on soft food. Briefly, all muscle-force vectors were derived from a combination of *in vivo* raw EMG signals collected when the animal was chewing on nuts and *ex vivo* muscle physiological cross-sectional area (PCSA) analysis to estimate the maximum force potential of each muscle (Panagiotopoulou et al., 2017). To assign the muscle-force vectors to our FEMs we selected surface nodes representing the insertion areas of the working- and balancing-side jaw-elevator muscles (deep and superficial masseter, anterior and posterior temporalis, medial pterygoid) determined from dissection pictures of the experimental subject. Force magnitude of each muscle was calculated by multiplying the estimated PCSA by 30 N/cm^2^, an estimate of the muscle-specific tension (Sinclair and Alexander, 1987), scaled to the mean normalized EMG amplitude at the time of maximum bone strain. The directional components of the force vectors were calculated using the cranial and mandibular attachments of each muscle, determined from dissection images of the experimental subject. All muscle force data are listed in Supplementary Table S1.

To simulate bite force, we constrained the occlusal surfaces of the working (left) side premolars and first molar and fixed them against translations in all directions (Panagiotopoulou et al., 2017). To allow for lateral transverse bending of the mandible we selected one node at the top of each mandibular condyle and fixed the right (balancing-side) condyle against translations in the anterior-posterior and superior-inferior directions but allowed medio-lateral translation. The working-side mandibular condyle was fixed against translation in all directions and rotation was permitted at both condyles (Panagiotopoulou et al., 2017). To prevent friction between internal/embedded structures within FEMs (e.g., PDL and internal surfaces of cortical bone), all adjacent surfaces were bound together with tie constraints. All FEMs were solved using the Abaqus CAE Simulia software v2016 default implicit direct static solver (Dassault Systémes, Vélizy-Villacoublay, France). Average solution time (4 processors and 8 tokens) was ~10 min per model.

### Model Validation

To validate our PDL and NO PDL models we compared strains from our FEMs to *in vivo* mandibular bone strains collected from the same individual while the animal was chewing on nuts. Experimental protocols for the collection and analysis of the *in vivo* strain gauge data are detailed elsewhere (Panagiotopoulou et al., 2017). Briefly, *in vivo* strain data were collected from three rosette strain gauges surgically bonded to the left (working side) mandible. Two were fixed to the buccal aspect of the working- side corpus (below the first molar, beside the most anterior attachment of the superficial masseter muscle), one close to the inferior border (LLAT) and the other close to the horizontal midline (ULAT). The third gauge (MED) was fixed to the lingual surface of the working-side corpus, inferior to the insertion of the mylohyoid muscle, beneath the left first molar. To locate the gauge sites in the FEM, Panagiotopoulou et al. (2017) overlayed FE mesh files with radiographs taken of the macaque head shortly after surgical implantation of the gauges, aligning them manually to achieve best fit. Strain tensors from the surface elements at these locations then were obtained and rotated to match the coordinate system of the corresponding strain gauge. These strain tensors were used to calculate the magnitude and direction of principal strains at the gauge locations in the FEMs, allowing for comparison to *in vivo* strain data. Raw *in vivo* strains from the strain gauge locations when the animal was chewing on nuts are provided in Supplementary Table S2.

### Testing Predictions

*Hypothesis 1: Inclusion of the PDL (and associated alveolar tissues), creating a more compliant interface between tooth and bone, decreases the torsional rigidity of the jaw and increases global surface strains; absence of the PDL (and associated alveolar tissues) increases the jaw's torsional rigidity and decreases global surface strains*.

*Prediction 1*: *When the PDL is included in FEMs of the jaw, progressive increases in its Young's Modulus (i.e., decreases in its compliance) will increase the torsional rigidity of the jaw, resulting in progressively lower global surface principal strain (*ε*1—maximum principal strain and* ε*2—minimum principal strain) magnitudes*.

To determine if increasing the PDL's Young's Modulus increases the jaw's torsional rigidity and progressively decreases principal strain magnitudes we plotted the maximum and minimum principal strains from homologous locations across the working-side (left) corpus in Models 1–4. We also calculated element level differences in principal strain magnitudes in the cortical bone of Models 1 and 4 and have outlined specific anatomic locations for comparison (Supplementary Figure S2). To calculate element level differences, we exported principal strains from Abaqus CAE Simulia (v2016), and used custom written MatLab code to calculate the element level difference in results and projected these values onto the 3D model of cortical bone.

*Prediction 2*: *The FEM of the jaw that excludes the PDL will experience lower global surface principal strain magnitudes during simulated unilateral post-canine chewing than the FEM including the PDL*.

To quantify differences in principal strains we calculated elemental differences in principal strains found in the cortical bone and trabecular bone tissue of the PDL and NO PDL models, using the same method outlined for *Prediction 1*.

*Prediction 3*: *The FEM of the jaw that includes the PDL will experience greater torsion of the balancing- and working-side corpora, and higher associated shear strains, than the FEM that excludes the PDL*.

To quantify whether excluding the PDL lowers shear strains associated with torsion of the working- and balancing-side corpus we calculated elemental differences in the sagittal-XY, frontal-XZ and transverse-YZ shear strains in the cortical bone.

*Hypothesis 2: The PDL acts to redirect masticatory stresses from the tooth roots to the alveolar bone*.

*Prediction: We predict that FEMs without the PDL will have higher strains in the tooth roots than FEMs with the PDL*.

To evaluate whether the PDL redirects masticatory stresses from the tooth roots to the alveolar bone we compared strains in the alveolar tissues (teeth and PDL) of the PDL and NO PDL models. We calculated elemental differences in principal (ε_1_ and ε_2_) and shear (sagittal-XY, frontal-XZ and transverse-YZ) strains found in the 3D geometries of the PDL and teeth.

## Results

### Model Validation

**Figure 1:**
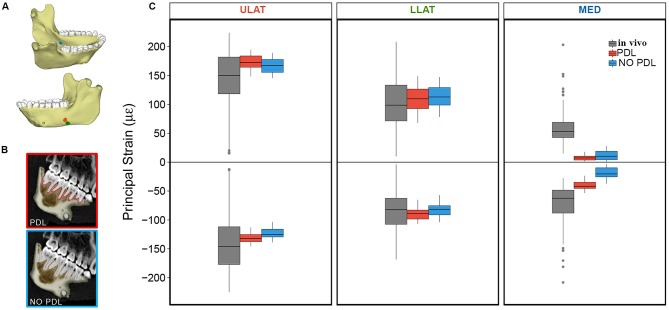
**(A)** Mandible model with markers indicating locations of the ULAT (red), LLAT (green), and MED (blue) strain gauge sites (modified from Panagiotopoulou et al., 2017: Figure 7). **(B)** CT scan slices of mandible indicating the breakdown of the PDL and NO PDL models. **(C)** Box plots of ε_1_ (positive) and ε_2_ (negative) principal strain magnitudes at the ULAT, LLAT, and MED gauge sites for PDL and NO PDL FEMs and the *in vivo* experiment. Center lines represent the median, upper and lower box boundaries represent 25th and 75th percentiles, respectively; upper and lower whiskers represent 1.5 × inter-quartile range; points are outliers.

**Figure 2:**
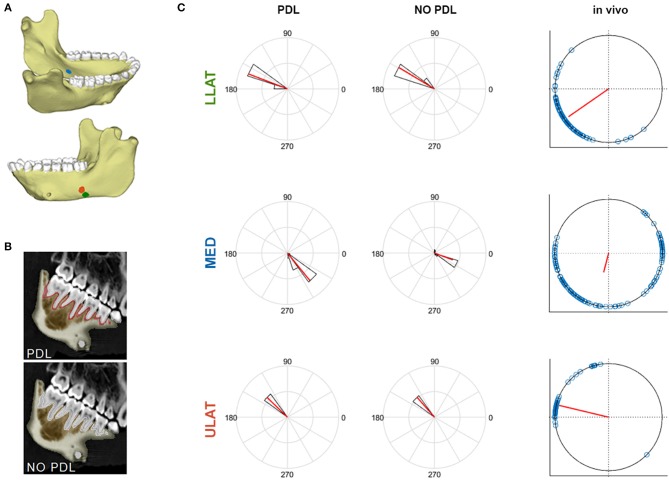
**(A)** Mandible model with markers indicating locations of the ULAT (red), LLAT (green), and MED (blue) strain gauge sites. **(B)** CT scan slices of mandible indicating the breakdown of the PDL and NO PDL models. **(C)** Polar histogram of the distribution of **ε_1_** orientations (in degrees) at the LLAT, ULAT, and MED gauge sites of PDL and NO PDL FEMs compared to the *in vivo* experiment. Red solid lines indicate the mean orientation. Black bars and blue circles represent the range of FEMs and *in vivo* data, respectively.

In two of the three gauge sites (ULAT and LLAT), median principal strain magnitudes from both the PDL and NO PDL FEMs are within 1.5^*^interquartile range (IQR) of the *in vivo* data (Figure 1). At the third gauge site, only the ε_2_ principal strains from the PDL model where within 1.5^*^IQR of the *in vivo* data (Table 1), though principal strains from both FEMs were within the broader range of those recorded *in vivo*. At all three gauge locations principal strain orientations from FEMs were within the range of those measured *in vivo* (Figure 2).

**Table 1:** Descriptive statistics for maximum (Max), minimum (Min), and mean principal (ε_1_ and ε_2_) strain magnitude (με) for the PDL and NO PDL FEMs, and the *in vivo* data.

			**ε_1_**				**ε_2_**				**ε_1_ Orientation**	
**Gauge location**	**Model**	***n* elements**	**Mean**	**SD**	**Min**	**Max**	**Mean**	**SD**	**Min**	**Max**	**Mean**	**SD**
ULAT	PDL	56	173	12	148	195	−131	8	−146	−113	68	1
	NO PDL	56	166	12	145	189	−123	9	−139	−103	66	2
	*In vivo*	148	150	61	15	271	−141	57	−249	−12	83	9
LLAT	PDL	54	109	21	68	149	−89	12	−107	−65	80	2
	NO PDL	54	113	18	78	147	−82	12	−104	−57	74	3
	*In vivo*	148	103	47	10	208	−84	37	−168	−4	287	12
MED	PDL	24	7	4	1	18	−40	8	−53	−24	335	5
	NO PDL	37	11	8	1	28	−19	9	−37	−3	350	19
	*In vivo*	148	59	27	15	203	−72	33	−208	−28	308	32

*Gauge locations: ULAT, LLAT, and MED are abbreviations for upper lateral, lower lateral, and medial (cf. Figure 1). SD represents standard deviation*.

*Hypothesis 1: Inclusion of the PDL (and associated alveolar tissues), creating a more compliant interface between tooth and bone, decreases the torsional rigidity of the jaw and increases global surface strains; absence of the PDL (and associated alveolar tissues) increases the jaw's torsional rigidity and decreases global surface strains*.

**Figure 3:**
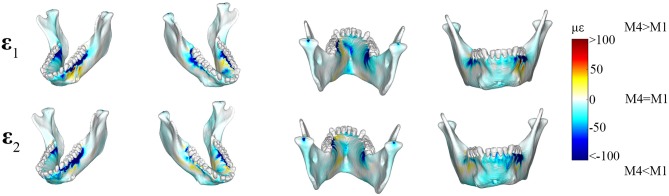
Color plots of differences in maximum (ε_1_) and minimum (ε_2_) principal strain magnitudes between Model 1 (PDL *E* = 0.07 MPa) and Model 4 (PDL *E* = 1,750 MPa). Pairs of FEMs are compared by mapping the cortical bone surface distribution of element level *differences* in principal strains between the two models onto the surfaces of the model. Panels compares **ε**_1_ and **ε**_2_ magnitudes between the model pairs in four views. Scale bars to the right of each panel indicate the difference in principal strains (με) between models. Areas of greater difference in results from Models 1 and 4 are indicated by darker colors, while lighter colors indicate areas of high similarity.

Principal strain comparisons in models that vary the Young's Modulus of the PDL (0.07–1,750 MPa) support *Prediction 1* such that increasing the Young's Modulus of the PDL decreases global principal strains (Supplementary Figure S3). Plotting the elemental differences of ε_1_ strain magnitudes between the FEMs with the extreme Young's moduli [Model 1 (*E* = 0.07 MPa) vs. Model 4 (*E* = 1,750 MPa)] showed that Model 4 had ε_1_ strains higher than Model 1 (>100 με) along the buccal aspect of the working-side corpus at the level of the premolars (Figure 3). Model 4 yielded lower (>100 με) ε_1_ strains than Model 1 at the alveoli of the post canine teeth of both the working- and balancing- sides and the lingual symphysis. In addition, Model 4 experienced marginally lower (~50 με) and considerably lower (>100 με) ε_2_ strains than Model 1 in the alveoli and in the labial symphysis (Figure 3).

**Figure 4:**
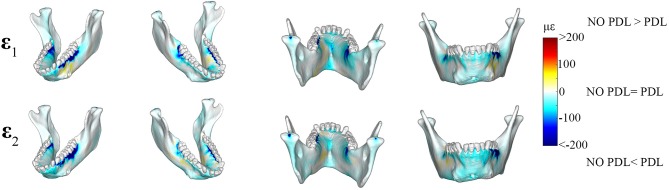
Color plots of differences in maximum (ε_1_) and minimum (ε_2_) principal strain magnitudes between models that include (PDL Model) and exclude (NO PDL Model) the periodontal ligament. Pairs of FEMs are compared by mapping the cortical bone surface distribution of element level *differences* in principal strains between the two models onto the surfaces of the model. Panels compare ε_1_ and ε_2_ magnitudes between the model pairs in four views. Scale bars to the right of each panel indicate the element level difference in principal strains (με) between models. Areas of greater difference in results from PDL and NO PDL models are indicated by darker colors, while lighter colors indicate areas of high similarity.

Plots of element-level differences between PDL and NO PDL cortical bone strains showed that *Prediction 2* was partially supported. Exclusion of the PDL resulted in lower principal (ε_1_ and ε_2_) strains on both the working- and balancing-side mandible (Table 2; Figure 4). However, at various mandibular sites the NO PDL model yielded ε_1_ magnitudes higher (diff. of 50–100 με) than the PDL model (Table 2; Figure 4)_._ Principal strain comparisons at the trabecular tissue level between the PDL and NO PDL FEMs showed similar patterns to cortical bone. The NO PDL model had trabecular bone ε_1_ and ε_2_ magnitudes lower than the PDL model in various locations across the mandible (Supplementary Table S3; Supplementary Figure S4) and ε_1_ magnitudes higher than the PDL in the trabecular bone of the alveoli (Supplementary Table S3; Supplementary Figure S4).

**Table 2:** Summary of differences in cortical bone of NO PDL and PDL models.

**Location**	**Direction of difference**	**Magnitude of difference**
**ε_1_–MAXIMUM PRINCIPAL STRAIN**		
**Working (left) side**		
Buccal alveoli, inferior to P_2_P_3_M_1_M_2_M_3_	NO PDL **<** PDL	>200 με
Lingual alveoli, inferior to P_2_P_3_M_1_M_2_	NO PDL **<** PDL	>200 με
Temporomandibular joint constraint point of condylar process	NO PDL **<** PDL	>200 με
Posterior aspect of buccal corpus	NO PDL **<** PDL	~50 με
Anterior aspect of buccal corpus	NO PDL **>** PDL	~50 με
Posterior aspect of lingual corpus	NO PDL **>** PDL	~50 με
**Balancing (right) side**		
Temporomandibular joint constraint point of condylar process	NO PDL **<** PDL	>200 με
Lingual and buccal alveoli inferior to M_1_M_2_	NO PDL **<** PDL	>200 με
Superior aspect of lingual corpus	NO PDL **<** PDL	~50 με
Retromolar region	NO PDL **>** PDL	~50 με
**Symphysis**		
Superior and inferior tori of lingual symphysis	NO PDL **<** PDL	~50 με
Inferior aspect of labial symphysis	NO PDL **<** PDL	~50 με
**ε_2_–MINIMUM PRINCIPAL STRAIN**		
**Working side (left) side**		
Buccal alveoli, inferior to P_2_P_3_M_1_M_2_M_3_	NO PDL **<** PDL	>200 με
Lingual alveoli, inferior to P_2_P_3_M_1_M_2_	NO PDL **<** PDL	>200 με
Superior aspect of lingual anterior corpus	NO PDL **<** PDL	~50 με
Inferior aspect of lingual ramus	NO PDL **>** PDL	~50 με
**Balancing side**		
Buccal and lingual corpus inferior to balancing side M_3_	NO PDL **<** PDL	>200 με
Inferior aspect of inferior		
Posterior aspect of ramus superiorly	NO PDL **<** PDL	~50 με
Lingual corpus	NO PDL **<** PDL	~50 με
Buccal posterior aspect of corpus	NO PDL **>** PDL	~ 50 με
**Symphysis**		
Lingual inferior and superior aspect	NO PDL **<** PDL	~50 με
Labial inferior and superior aspect	NO PDL **<** PDL	~50 με

**Figure 5:**
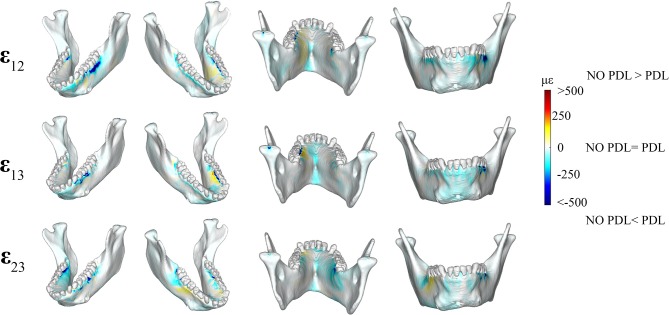
Color plots of differences in shear strain magnitudes between models that include (PDL Model) and exclude (NO PDL Model) the periodontal ligament. Pairs of FEMs are compared by mapping the cortical bone surface distribution of element level *differences* in shear strains between the two models onto the surfaces of the model. Panels compare **ε**_12_ (sagittal), **ε**_13_ (frontal), and **ε**_23_ (transverse) shear strain magnitudes between the model pairs in four views. Scale bars to the right of each panel indicate the difference in strains (με) between models. Areas of greater difference in results from PDL and NO PDL models are indicated by darker colors, while lighter colors indicate areas of high similarity.

Shear strain comparisons between the PDL and NO PDL models supported *Prediction 3* that exclusion of the PDL in FEA reduces torsion in the balancing-and working-side corpora (Figure 5).

We observed lower frontal (ε_13_) shear strains in the NO PDL model in the anterior corpus (decrease of ~100 με) (Figure 5) and in the alveolar bone of the balancing- side M_3_ and working-side P_3_P_4_M_1_ (decrease of more than 500 με). We found higher frontal shear strains (~100 με) in the NO PDL model at the working-side alveoli of P_3_P_4_M_1_ lingually.

We measured lower (>500με) transverse (ε_23_) shear strains in the NO PDL than the PDL model (Figure 3), except for slightly higher (~100 με) transverse shear strains in the NO PDL model at the anterior corpus of the balancing side (Figure 5).

Sagittal shear strains (ε_12_) were lower (~100 με) in the NO PDL model in the alveolar bone and the lingual symphysis (Figure 5). Particularly large decreases in ε_12_ (<500 με) were found in the alveolar bone below the working side molars and balancing side M_3_ (Figure 5). However, sagittal shear strains were higher in the NO PDL model in the working-side lingual anterior corpus (Figure 5).

All raw data used in Table 2 and to generate Figures 3–5; Supplementary Figures S3, S4 are available at figshare (Mehari Abraha et al., 2019).

*Hypothesis 2: The PDL acts to redirect masticatory stresses from the tooth roots to the alveolar bone*.

**Figure 6:**
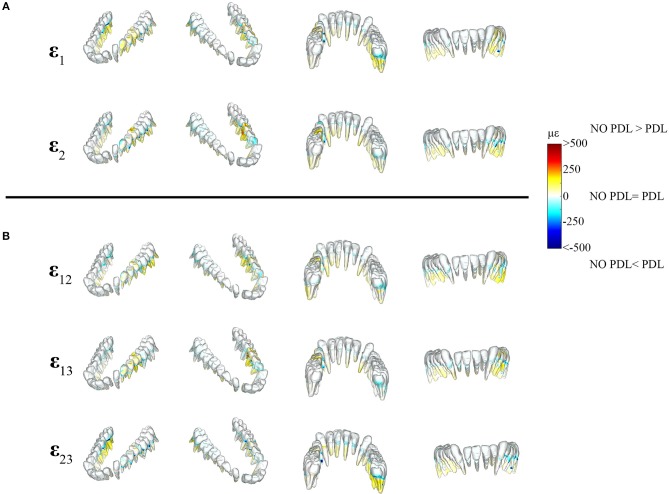
Color plots of differences in strain magnitudes found in the teeth of FEMs that include (PDL Model) and exclude the periodontal ligament (NO PDL Model). Pairs of FEMs are compared by mapping the cortical bone surface distribution of element level *differences* in principal strains between the two models onto the surfaces of the teeth. **(A)** Shows differences in ε_1_ and ε_2_ principal strains magnitudes. **(B)** Shows differences in sagittal (ε_12_), frontal (ε_13_), and transverse (ε_23_) shear strain magnitudes between model pairs in four views. Scale bars to the right of each panel indicate the element level difference in strains (με) between models. Areas of greater difference in results from PDL and NO PDL models are indicated by darker colors, while lighter colors indicate areas of high similarity.

**Figure 7:**
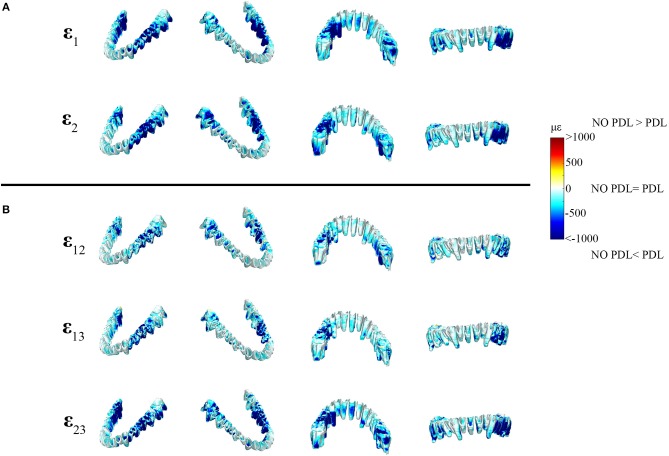
Color plots of differences in strain magnitudes found in the periodontal ligament of FEMs that include (PDL Model) and exclude (NO PDL Model) the PDL. Pairs of FEMs are compared by mapping the surface distribution of element level *differences* in principal strains between the two models onto the teeth and the models are shown in four views. **(A)** Shows differences in ε_1_ and ε_2_ principal strains magnitudes. **(B)** Shows differences in sagittal (ε_12_), frontal (ε_13_), and transverse (ε_23_) shear strain magnitudes between model pairs in four views. Scale bars to the right of each panel indicate the element level difference in strains (με) between models. Areas of greater difference in results from PDL and NO PDL models are indicated by darker colors, while lighter colors indicate areas of high similarity.

The differences between principal strains of PDL and NO PDL models in the alveolar tissues (teeth and PDL) were the largest recorded in the entire FEM (diff. >1,000 με) (Figures 6, 7 vs. Figure 4), demonstrating that at a local level, excluding the PDL increased strains in the tooth roots and decreased strains in the PDL. Thus, our prediction that FEMs without the PDL will have higher strains in the tooth roots than FEMs with the PDL is supported.

Detailed analysis of principal strains in the dentition showed that in the tooth roots of balancing- and working-side post canine teeth the NO PDL model had higher (increase of >~250 με) ε_1_ and ε_2_ strains than the PDL model (Figure 6). The greatest increase in ε_2_ strains was found in the first molar on the working side (difference >~500 με) (Figure 6). There were some locations (e.g., tooth-bone junction point) in the balancing- and working-side molars where the NO PDL model had lower ε_1_ and ε_2_ strains (decrease of >~500 με) (Figure 6). The NO PDL model also experienced higher sagittal (XY) shear strain in the roots of the working side P_3_P_4_M_1_, higher frontal (XZ) shear strain in the roots of the working side molars (increase of >~250 με) and higher transverse (YZ) shear strain (increase of >~250 με) in the roots of the balancing side molars (Figure 6).

In the periodontal ligament itself, the NO PDL model experienced lower ε_1_ and ε_2_ strains (decrease of >500 με) than the PDL model (Figure 7). Specifically, the ligament tissue surrounding the working-side premolars and first molar, and the balancing-side third molar had ε_1_ strains that were higher than the NO PDL model by more than 500 με (Figure 7). Similarly, ε_2_ strains were more than ~500 με higher in the PDL model in the ligament tissue of the working-side premolars and first molar and the balancing-side third molar (Figure 7). The NO PDL model also had sagittal (XY), frontal (XZ) and transverse (YZ) shear strains more than 1,000 με lower in the periodontal ligament surrounding the working-side post-canine dentition and the balancing-side molars (Figure 7).

All raw data used to generate Figures 6, 7 are available at figshare (Mehari Abraha et al., 2019)

## Discussion

### Comparison of Results With Previous Studies of the PDL

While our findings partially confirm the hypothesis that the presence of the PDL decreases the torsional rigidity of the jaw and affects global surface strains, the degree to which our results are consistent with existing literature varies. Gröning et al. (2011) compared a PDL FEM (*E* = 0.01 MPa) to a NO PDL FEM where the PDL was modeled as cortical bone (*E* = 17 000 MPa) and found that excluding the PDL decreased global surface principal strains. However, we did not find the same degree of variation between our PDL and NO PDL FEMs. When Gröning et al. (2011) contrasted PDL and NO PDL FEMs, they found ε_1_ differences ≥500 με in several areas in the cortical bone, namely in the labial symphysis (ε_1_ was 543 με higher in PDL model) and below the post-canine dentition on the balancing side (ε_1_ was 519 με higher and ε_2_ was 546 με lower in the PDL model). In contrast, the greatest degree of variation noted in our study was in the periodontal tissue (diff. >1,000 με–Figure 7), and only smaller differences were found elsewhere in the mandible (Table 2; Figure 4).

Differences between our results and those of Gröning et al. may be attributed to differences in the boundary conditions of the FEMs (Gröning and Fagan, 2012; Grosse et al., 2012). While we allowed medio-lateral translation of one condyle to allow for lateral transverse bending of the jaw (Hylander, 1984, 1985; Panagiotopoulou et al., 2017), Gröning et al. (2011) fixed both condyles against translation in all directions using a compliant pad of soft tissue. In their study (Gröning et al., 2011) conducted sensitivity analysis to determine whether constraining the jaw through a compliant pad of soft tissue influenced results, and found that constraining the condyles directly did not affect their findings (i.e., the presence of the PDL still had a large effect on local and global principal strains). However, to the best of our knowledge, they did not run a model where mediolateral translation of one condyle was allowed. To test whether these condylar constraints affected model sensitivity to the PDL, we over-constrained our PDL and NO PDL models (i.e., condyles constrained in all directions) and compared bone strain regimes. We found no increased sensitivity to the presence of the PDL (i.e., no pronounced change to our results) (Supplementary Figure S5).

Disparity between our results and those of Gröning et al. (2011) could also be due to variation in the directional forces and the co-activation of the adductor jaw muscles. While our model applied muscle forces associated with unilateral post-canine chewing (based on *in vivo* and subject-specific experiments), Gröning et al. (2011) applied muscle force vectors associated with unilateral molar clenching (derived from Nelson, 1986; Korioth et al., 1992; Korioth and Hannam, 1994; van Eijden et al., 1995, 1996). To test whether different feeding behaviors (post-canine chewing vs. clenching) could influence the difference in strain regimes between the PDL and NO PDL models we re-ran our models using the muscle activation scaling of the major jaw-closing muscles provided in Gröning et al. (2011), originally reported in Nelson (1986). Though we were unable to include the directional component of the muscle force vectors used by Gröning et al. (2011) and we did not include the inferior head of the lateral pterygoid muscle, we found that assigning muscle forces associated with clenching did result in a greater sensitivity of our FEM to the presence/absence of a PDL (Supplementary Figure S6). Considering these additional findings, we propose that variations in the activation peaks of the major muscles of mastication in clenching vs. chewing behaviors, particularly the working- and balancing-side superficial masseters and medial pterygoids, may have altered the twisting moments of the corpora and in turn, affected the FEMs sensitivity to the presence of the PDL. This coupled with variations in mandibular morphology and masticatory musculature between humans and macaques, may account for the discrepancies between our results and those of Gröning et al. (2011).

Wood et al. (2011), in contrast to Gröning et al. (2011), found that excluding the PDL resulted in lower principal strains in the canine and molar alveolar regions of the cranium. However, Wood et al. (2011) found “negligible” differences elsewhere in the mandible, while we found differences of ~200 με. Divergence between our results and those of Wood et al. (2011) could be attributed to differences in the size of the dentition relative to the skeletal element (cranium vs. mandible), as larger tooth roots would increase the jaws resistance to torsional deformation and decrease a jaw FEMs sensitivity to the PDL. However, such incongruity could also be attributed to differences in the methods of comparison between FEM strain regimes. The differences between PDL and NO PDL models in our study are difficult to distinguish when contrasting color plots of maximum and minimum principal strains across the mandible (Supplementary Figures S7, S8) but are much easier to identify in element differential plots between models (Figure 4). Hence, while Wood et al. (2011) report negligible differences between models that include and exclude the PDL in areas away from the alveoli, it is possible that with element level comparisons these differences may become more apparent.

We observed that excluding the PDL from the FEMs decreased strains in the alveolar bone but increased strains in the tooth roots (Figures 6, 7). These results broadly support the hypotheses that the PDL transmits/redistributes strains across the bone-PDL-tooth interface (McCormack et al., 2017; Nikolaus et al., 2017) while also providing further credibility to the specific hypothesis proposed by Nikolaus et al. (2017) that under masticatory loads the PDL acts to shield the tooth roots by redirecting strains to the alveolar bone. Their hypothesis was previously supported by high resolution, small scale testing of strains in sections of the human and pig mandibular corpora (McCormack et al., 2017; Nikolaus et al., 2017). However, our findings highlight that even in large scale FEA (where the PDL is modeled as a simplified, linear elastic tissue) the PDL's mechanical effect on the local strain environment is still evident.

### Implications for Modeling the PDL in FEA

Overall, our results demonstrate that the PDL has some effect on the global surface maximum and minimum principal strains in the macaque mandible during unilateral post-canine chewing. Yet it is worth noting that comparisons of corpus surface strains between the FEMs (with and without the PDL) to *in vivo* strain gauge data showed that both FEMs yield strain magnitudes and orientations within the range of *in vivo* bone strains (Figures 1, 2) (Panagiotopoulou et al., 2017). Thus, whilst the PDL tissue has some localized effect on strain magnitudes across the jaw when included in FEA, it does not appear to substantially alter the mechanical behavior of the jaw during unilateral post-canine chewing. Therefore, if the hypotheses being tested relate to large scale differences in the magnitude/orientation/patterns of principal strains across individuals or species under these loading conditions, it is unlikely that excluding the PDL or varying its Young's Modulus within the published range of 0.07–1,750 MPa will have a large effect on research findings. If, however, the hypotheses are concerned with small-scale differences in principal strains between models (e.g., element-to-element comparison) then variation in the Young's Modulus of the PDL and/or exclusion of the PDL tissue may result in strain differences, and sensitivity analysis is required.

A limitation of our study is that we tested the effect of the PDL under very specific masticatory loading conditions (post-canine chewing on nuts). As outlined by Hylander (1979) the position of the bite point may affect torsion of the corpora. For example, bite forces that pass lateral to the longitudinal axis of the corpora may result in twisting the corpus such that the inferior border inverts and the alveolar process everts, while forces that pass medial to the longitudinal axis may do the opposite (Hylander, 1979). Further, our study was limited to a single species (*M. mulatta)* and as discussed previously (Daegling and Hylander, 1998; Daegling, 2007), different corporal geometries across species may affect the torsional rigidity of the jaw. Given that any effect on mandibular torsion may affect a FEMs sensitivity to the PDL, further sensitivity studies (using a combination of *in vivo* data collection and computer modeling) testing other loading conditions (e.g., incisor biting, unilateral biting) and/or other primate species are needed (Picton and Wills, 1978; Mow et al., 1984; Dorow et al., 2003; Natali A. et al., 2004; Wang et al., 2012; Sandino et al., 2015).

Our study did not account for the effect of variation in the PDL's thickness on local/global surface strain regimes. A previous small-scale FEA study captured the geometry of a molar tooth-PDL-bone complex from a pig mandible using μCT scans (pixel resolution 0.012 mm) and found that the PDL's thickness varied between 0.05 and 0.5 mm from region to region within the tooth root (median 0.21 mm; Nikolaus et al., 2017). However, in the case of mesh-based FEA, automatic non-manifold mesh generation requires a minimum thickness of 3 voxels for each structure, as fewer voxels causes unresolvable overlapping and intersecting triangles. Thus, accurately capturing variation in PDL thickness (~0.05–0.5 mm) would require a scan resolution where pixel size is ~0.016 mm, which was outside the range of our scans (0.2 mm).

Lastly, our study focused on the effect of the PDL when modeled with simplicity in a static linear simulation of the entire mandible. Modeling the PDL as a time-dependent tissue would likely provide us with greater insight on the mechanical function of the PDL during chewing; however, it would require the creation of a dynamic FEM, which was outside the scope of this study. Modeling the PDL as a hyperelastic tissue (e.g., Wood et al., 2011; Nikolaus et al., 2017) in a static simulation requires the definition of load- and tissue-specific material parameters, and validation and calibration of these parameters using load- and tissue- specific experimental data. While we did not include a FEM in which the PDL is modeled as a hyperelastic or viscoelastic tissue, we can note that Wood et al. (2011) compared a static FEM where the PDL was included and modeled as linear elastic (*E* = 0.68 MPa, *v* = 0.49) to a static FEM where the PDL was modeled as a Mooney Rivlin hyper-elastic material, with parameters derived from Genna et al. (2007). They also compared a dynamic model where the PDL was assigned linear elastic properties to a dynamic model where the PDL was modeled as viscoelastic (using the Maxwell chain model outlined in Natali A. et al. (2004). They found that that inclusion of hyperelastic or viscoelastic material properties for the PDL in the respective static and dynamic simulations had no visible effect on global surface strain regimes in areas away from the loaded teeth.

## Data Availability Statement

The datasets (results from finite element models) for this study can be found at Monash figshare (Mehari Abraha et al., 2019).

## Ethics Statement

Ethical review and approval was not required as this was a computer-based study. All *in vivo* data used in this study were collected as part of a previous publication (Panagiotopoulou et al., 2017).

## Author Contributions

HM, OP, CR, and AT designed the study. OP built and solved the PDL FEM. HM built and solved the NO PDL FEM. HM, JI-D, and OP contributed into the numerical analysis of the FEMs. HM wrote the manuscript with critical analysis and editing by OP, JI-D, AT, and CR.

### Conflict of Interest

The authors declare that the research was conducted in the absence of any commercial or financial relationships that could be construed as a potential conflict of interest.

## Supplementary Material

Supplementary Table S1 Muscle force data assigned to all finite element models for the major muscle of mastication, based on experimental data when the subject was chewing on nuts. The table shows the direction of the muscle action unit vector and estimated muscle force magnitude (rms EMG multiplied by the PCSA data × 30 kN/m^2^) for each muscle. Estimated muscle force was measured at the time of the maximum strain magnitude, measured from a gauge fixed to the labial surface of the inferior left mid-corpus (Panagiotopoulou et al., 2017).

Supplementary Table S2 Raw *in vivo* experimental data when the subject was chewing on nuts (almond, cashew, brazil nut, pecan, walnut, seeds).

Supplementary Table S3 Summary of differences in trabecular bone NO PDL and PDL models.

Supplementary Figure S1 CT scan slices depicting segmentation of the PDL, with schematic representation of the Young's Moduli assigned to the FEMs. In the PDL model the PDL is assigned Young's Moduli *(E)* of 0.68 MPa, while in the NO PDL model the PDL is assigned *E* value of 24,500 MPa (the same E value assigned to the teeth).

Supplementary Figure S2 Locations across the primate jaw where key differences between the PDL and NO PDL FEM were identified.

Supplementary Figure S3 Scatter plot of maximum ε_1_ (positive) and ε_2_ (negative) strains **(A)** found at sample locations **(B)** in Models 1–4, where the PDL is assigned Young's Moduli (*E*) of 0.07, 0.18, 13.8, and 1,750 MPa, respectively.

Supplementary Figure S4 Color plots of differences in maximum (ε_1_) and minimum (ε_2_) principal strain magnitudes in trabecular tissue between models that include (PDL Model) and exclude (NO PDL Model) the periodontal ligament. Pairs of FEMs are compared by mapping the surface distribution of element level *differences* in principal strains between the two models onto the trabecular tissue surface of the model. Panels compare ε_1_ and ε_2_ magnitudes between the model pairs in four views. Scale bars to the right of each panel indicate the element level difference in principal strains (με) between models. Areas of greater difference in results from PDL and NO PDL models are indicated by darker colors, while lighter colors indicate areas of high similarity.

Supplementary Figure S5 Comparisons of principal strain magnitudes in cortical bone of FEM model pairs (PDL vs. NO PDL), where both left and right TMJs are constrained against translation in all directions. Pairs of FEMs are compared by mapping the *differences* in principal strains between the two models onto the cortical surface of the model. Each panel compares ε_1_ and ε_2_ magnitudes between the model pairs in three views. Scale bars to the right of each panel indicate the difference in principal strains (με) between models. Areas of greater difference in results from PDL and NO PDL models are indicated by darker colors, while lighter colors indicate areas of high similarity.

Supplementary Figure S6 Comparisons of principal strain magnitudes in cortical bone of FEM model pairs (PDL vs. NO PDL), where models were loaded with muscle activation scale factors used in Gröning et al. (2011). Pairs of FEMs are compared by mapping the *differences* in principal strains between the two models onto the cortical surface of the model. Each panel compares ε_1_ and ε_2_ magnitudes between the model pairs in three views. Scale bars to the right of each panel indicate the difference in principal strains (με) between models. Areas of greater difference in results from PDL and NO PDL models are indicated by darker colors, while lighter colors indicate areas of high similarity.

Supplementary Figure S7 Color distribution of ε_1_ (maximum principal) strains of the whole FEM and trabecular tissue from PDL and NO PDL FEMs. Red and green ends of the scale represent higher and lower ε_1_ concentrations, respectively.

Supplementary Figure S8 Color distribution of ε_2_ (minimum principal) strains of the whole FEM and trabecular tissue from PDL and NO PDL FEMs. Blue and green ends of the scale represent higher and lower ε_1_ concentrations, respectively.
