# Supplementary material for: The Mechanical Effect of the Periodontal Ligament on Bone Strain Regimes in a Validated Finite Element Model of a Macaque Mandible
Source: Front Bioeng Biotechnol. 2019 Oct 30;7:269. doi: 10.3389/fbioe.2019.00269 (PMC6831558; doi:10.3389/fbioe.2019.00269)
Supplement: Supplementary Table S3 — Summary of differences in trabecular bone NO PDL and PDL models. [file Table_3.docx]

**Supplementary Table S3**: Summary of differences in trabecular bone NO PDL and PDL models

| Locations | **Direction of Difference** | **Magnitude of difference** |
| --- | --- | --- |
| ε_1_ – Maximum Principal strain |  |  |
| **Working Side** |  |  |
| interface between bone and P_2_P_3_M_1_ | NO PDL **<** PDL | greater than 200µε |
| posterior buccal corpus | NO PDL **>** PDL | approx. 50µε |
| anterior buccal corpus | NO PDL **>** PDL | approx. 50µε |
| buccal corpus inferior to M_1_ | NO PDL **>** PDL | approx. 50µε |
| **Balancing Side** |  |  |
| buccal and lingual aspect of alveolar sockets of M_1_M_2_M_3_ | NO PDL **<** PDL | greater than 200µε |
| furcation point of M_1_M_2_M_3_ | NO PDL **>** PDL | greater than 200µε |
| posterior buccal corpus | NO PDL **>** PDL | approx. 50µε |
| lingual corpus | NO PDL **>** PDL | approx. 50µε |
| **Symphysis** |  |  |
| labial and lingual aspect of symphysis ­ | NO PDL **<** PDL | approx. 50µε |
| ε_2_ – Minimum Principal strain |  |  |
| **Working Side** |  |  |
| interface between bone and P_2_P_3_M_1_ | NO PDL **<** PDL | greater than 200µε |
| interface between bone and M_2_M_3_ | NO PDL **>** PDL | greater than 200µε |
| superior aspect of lingual corpus | NO PDL **<** PDL | approx. 50µε |
| inferior aspect of anterior lingual corpus | NO PDL **>** PDL | approx. 50µε |
| **Balancing Side** |  |  |
| buccal and lingual aspect of alveolar sockets of M_2_M_3_ | NO PDL **<** PDL | greater than 200µε |
| furcation point of M_1_M_2_M_3_ | NO PDL **>** PDL | approx. 50µε |
| posterior buccal corpus | NO PDL **<** PDL | approx. 50µε |
| **Symphysis** |  |  |
| lingual and labial symphysis | NO PDL **<** PDL | approx. 50µε |
