# Supplementary figures and images for: The Mechanical Effect of the Periodontal Ligament on Bone Strain Regimes in a Validated Finite Element Model of a Macaque Mandible
Source: Front Bioeng Biotechnol. 2019 Oct 30;7:269. doi: 10.3389/fbioe.2019.00269 (PMC6831558; doi:10.3389/fbioe.2019.00269)

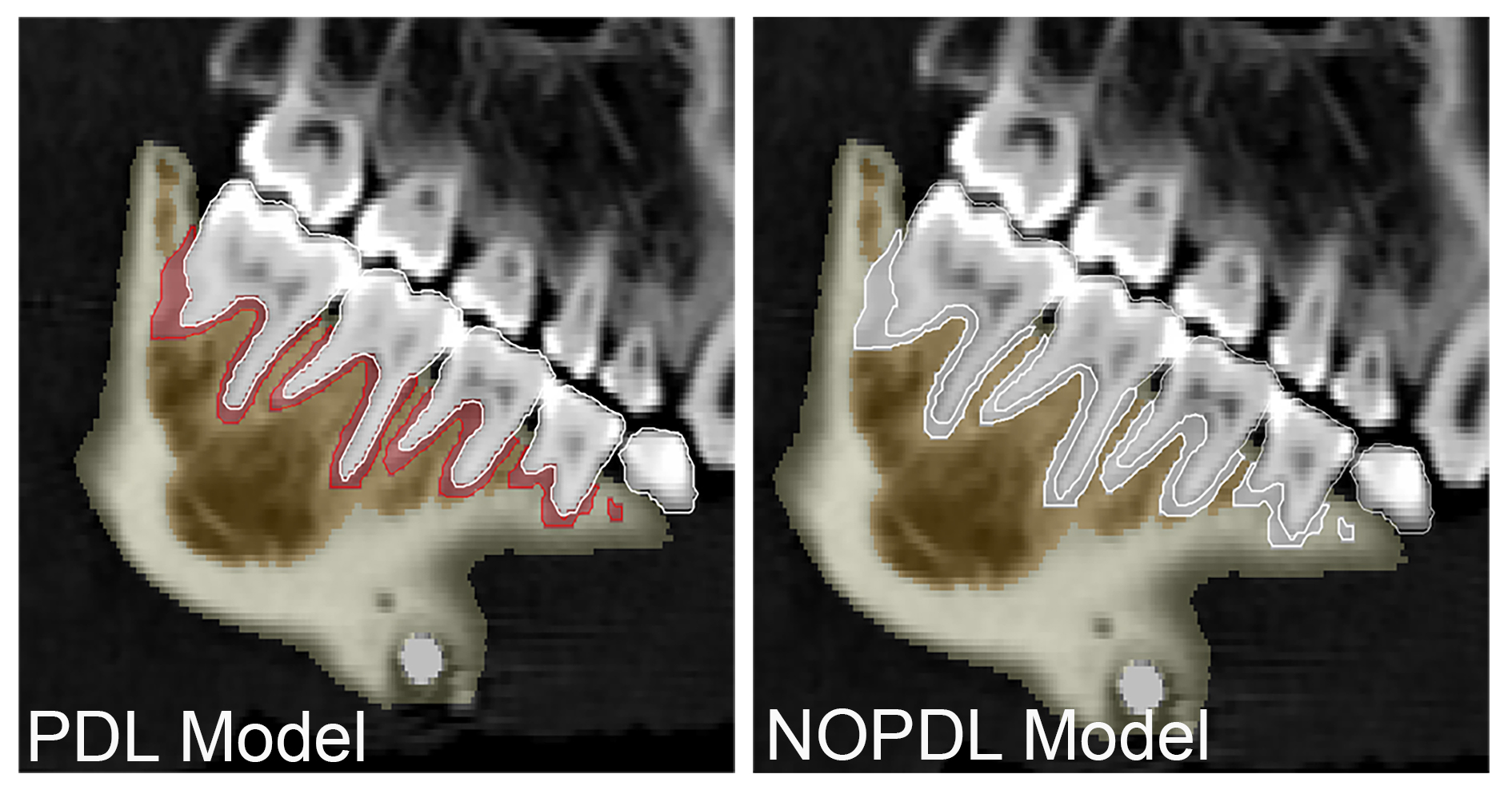

Supplement: Supplementary Figure S1 — CT scan slices depicting segmentation of the PDL, with schematic representation of the Young's Moduli assigned to the FEMs. In the PDL model the PDL is assigned Young's Moduli (E) of 0.68 MPa, while in the NO PDL model the PDL is assigned E value of 24,500 MPa (the same E value assigned to the teeth). [file Image_1.JPEG]

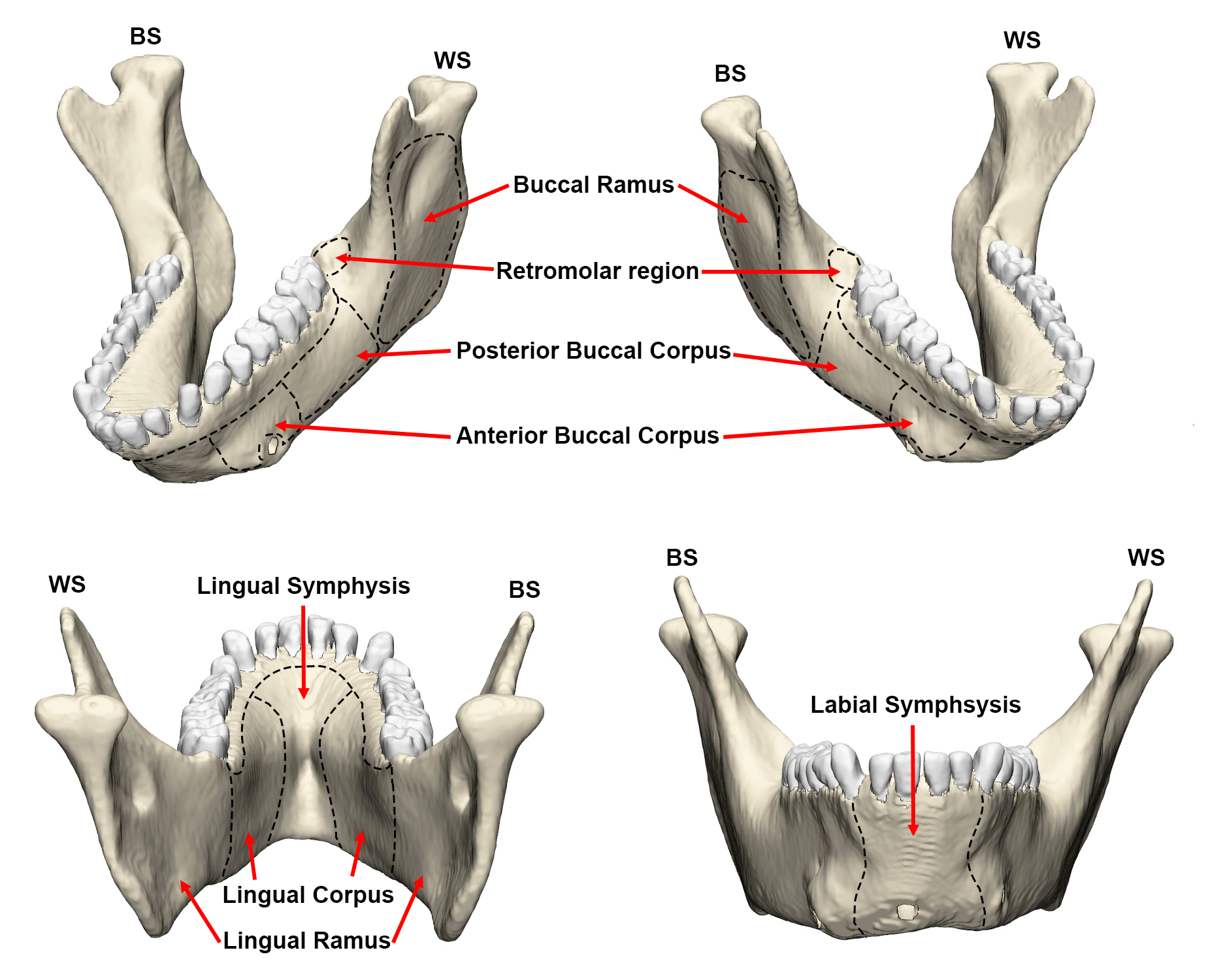

Supplement: Supplementary Figure S2 — Locations across the primate jaw where key differences between the PDL and NO PDL FEM were identified. [file Image_2.PNG]

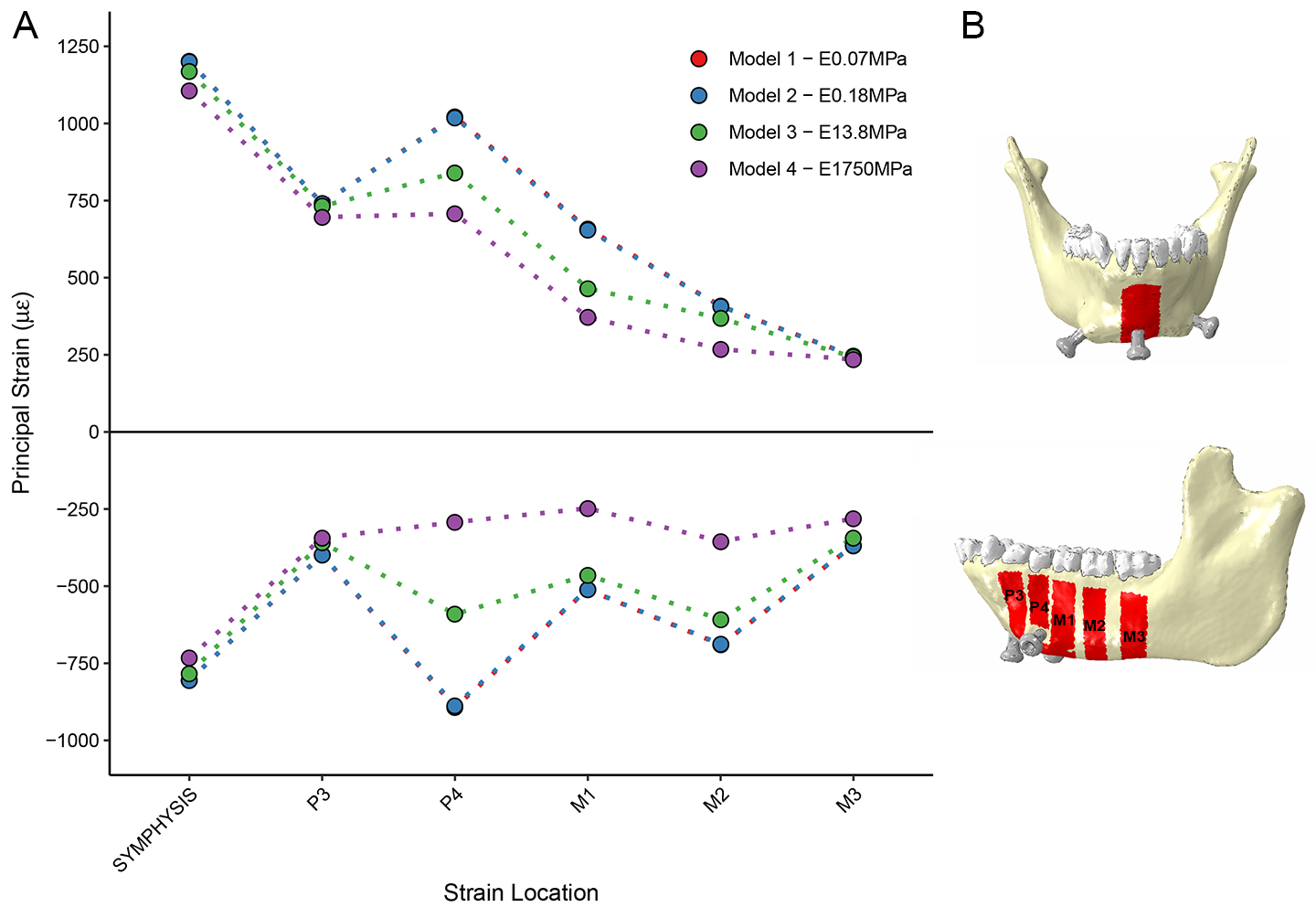

Supplement: Supplementary Figure S3 — Scatter plot of maximum ε1 (positive) and ε2 (negative) strains (A) found at sample locations (B) in Models 1–4, where the PDL is assigned Young's Moduli (E) of 0.07, 0.18, 13.8, and 1,750 MPa, respectively. [file Image_3.JPEG]

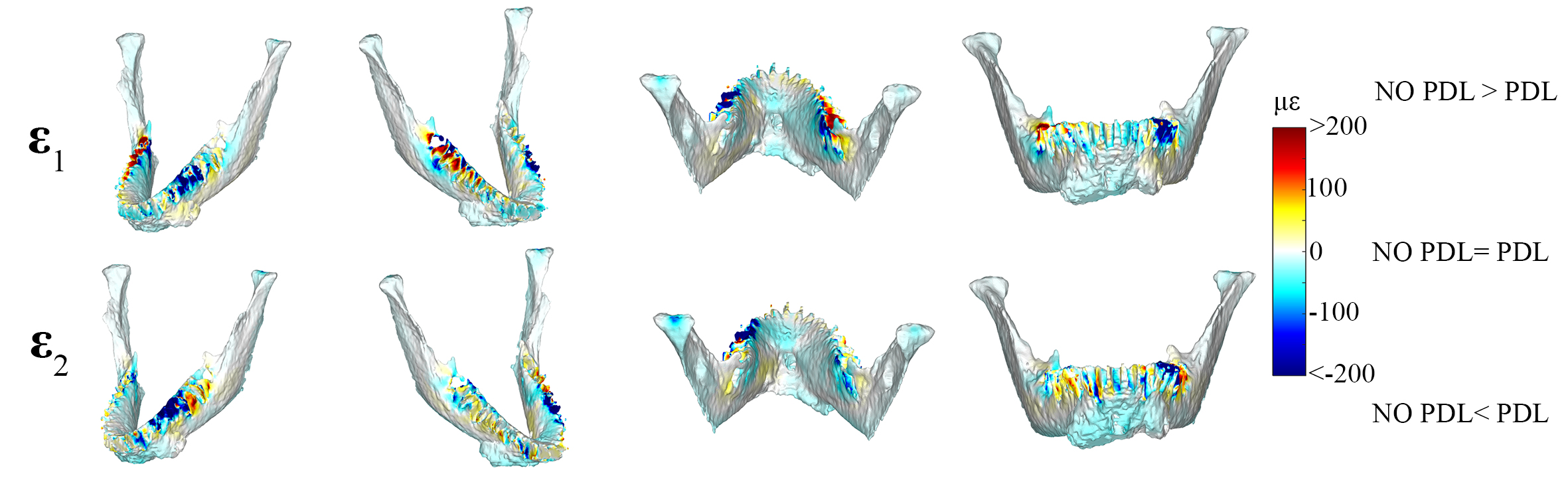

Supplement: Supplementary Figure S4 — Color plots of differences in maximum (ε1) and minimum (ε2) principal strain magnitudes in trabecular tissue between models that include (PDL Model) and exclude (NO PDL Model) the periodontal ligament. Pairs of FEMs are compared by mapping the surface distribution of element level differences in principal strains between the two models onto the trabecular tissue surface of the model. Panels compare ε1 and ε2 magnitudes between the model pairs in four views. Scale bars to the right of each panel indicate the element level difference in principal strains (με) between models. Areas of greater difference in results from PDL and NO PDL models are indicated by darker colors, while lighter colors indicate areas of high similarity. [file Image_4.JPEG]

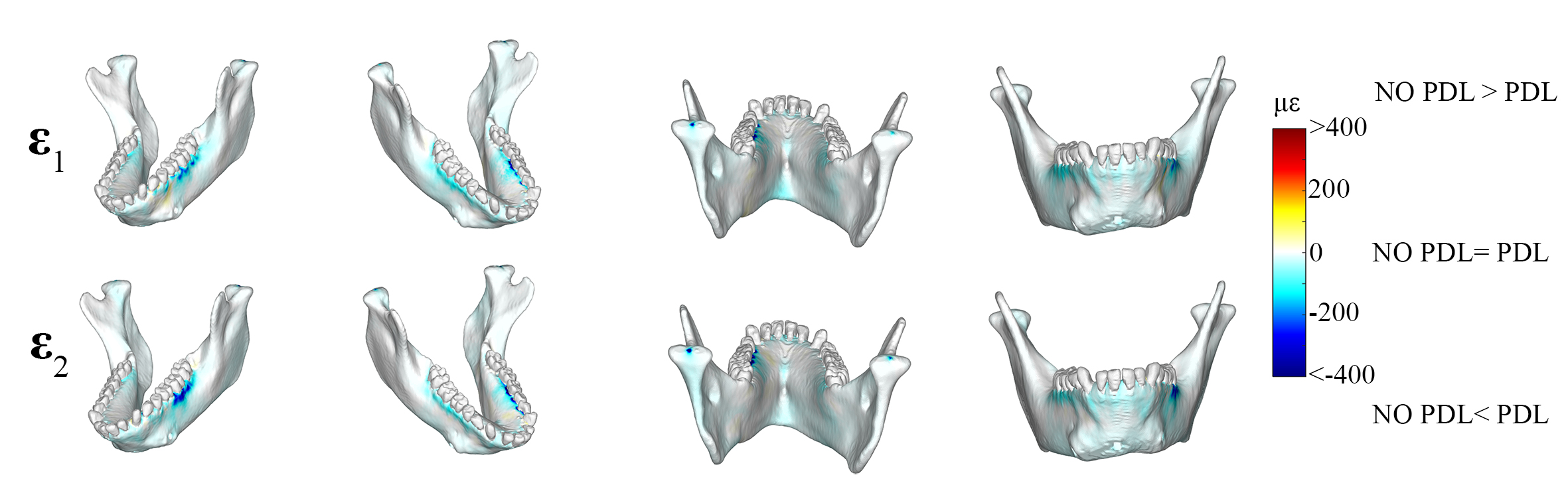

Supplement: Supplementary Figure S5 — Comparisons of principal strain magnitudes in cortical bone of FEM model pairs (PDL vs. NO PDL), where both left and right TMJs are constrained against translation in all directions. Pairs of FEMs are compared by mapping the differences in principal strains between the two models onto the cortical surface of the model. Each panel compares ε1 and ε2 magnitudes between the model pairs in three views. Scale bars to the right of each panel indicate the difference in principal strains (με) between models. Areas of greater difference in results from PDL and NO PDL models are indicated by darker colors, while lighter colors indicate areas of high similarity. [file Image_5.JPEG]

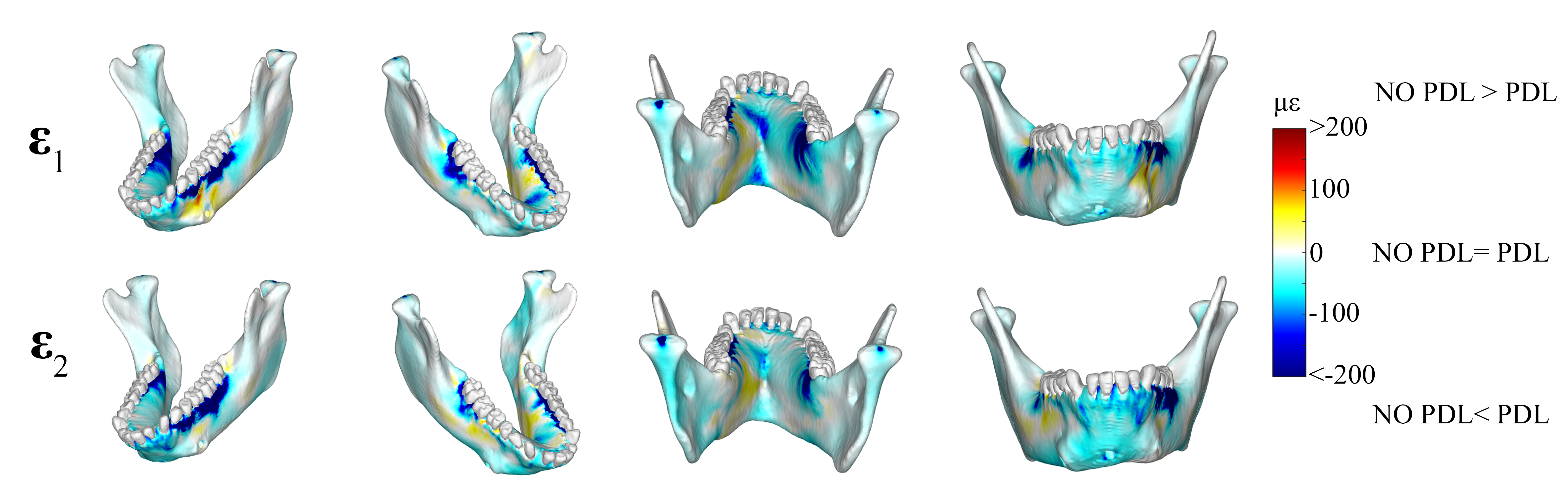

Supplement: Supplementary Figure S6 — Comparisons of principal strain magnitudes in cortical bone of FEM model pairs (PDL vs. NO PDL), where models were loaded with muscle activation scale factors used in Gröning et al. (2011). Pairs of FEMs are compared by mapping the differences in principal strains between the two models onto the cortical surface of the model. Each panel compares ε1 and ε2 magnitudes between the model pairs in three views. Scale bars to the right of each panel indicate the difference in principal strains (με) between models. Areas of greater difference in results from PDL and NO PDL models are indicated by darker colors, while lighter colors indicate areas of high similarity. [file Image_6.JPEG]

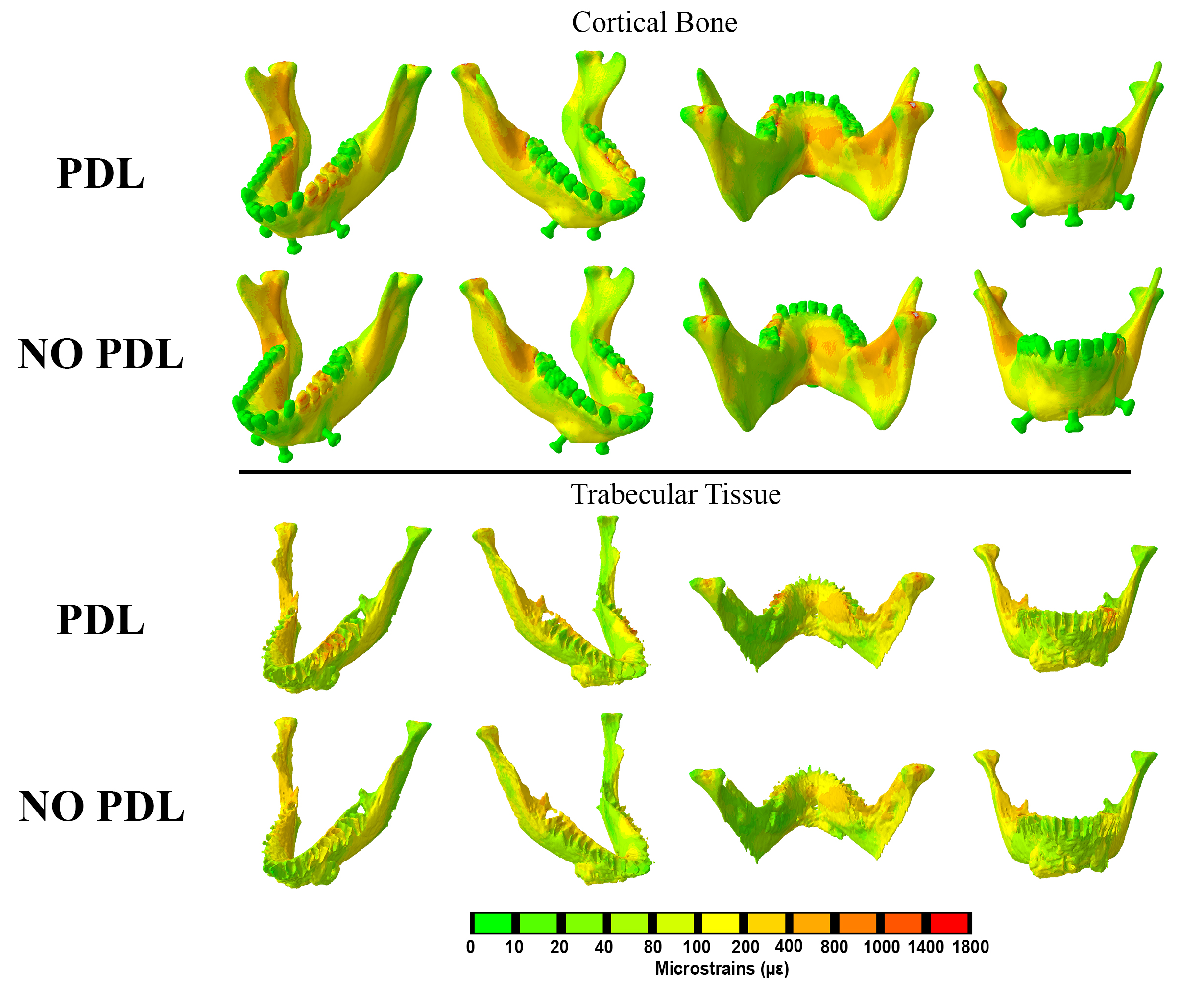

Supplement: Supplementary Figure S7 — Color distribution of ε1 (maximum principal) strains of the whole FEM and trabecular tissue from PDL and NO PDL FEMs. Red and green ends of the scale represent higher and lower ε1 concentrations, respectively. [file Image_7.JPEG]

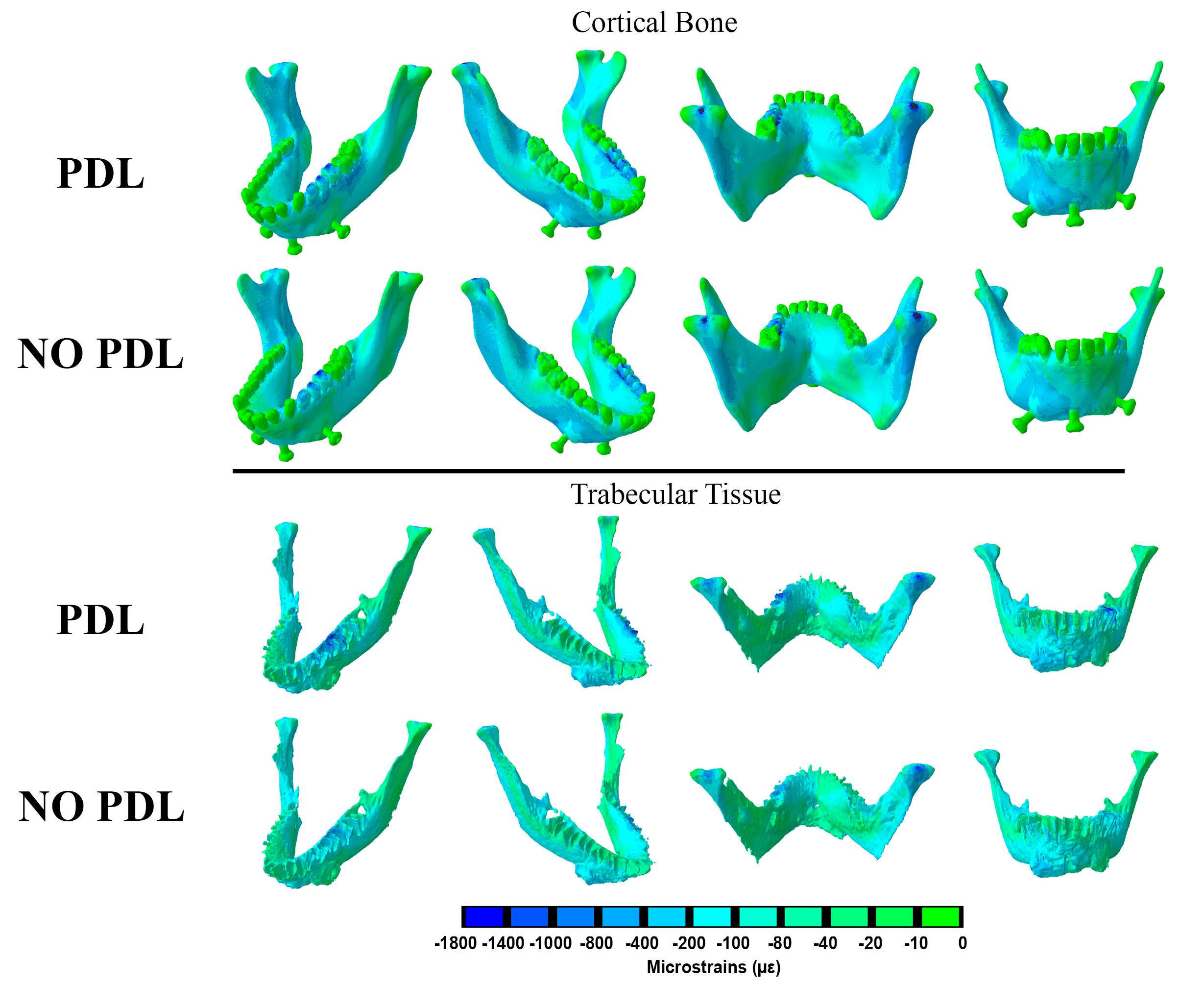

Supplement: Supplementary Figure S8 — Color distribution of ε2 (minimum principal) strains of the whole FEM and trabecular tissue from PDL and NO PDL FEMs. Blue and green ends of the scale represent higher and lower ε1 concentrations, respectively. [file Image_8.JPEG]
